# Low-dose radiation cancer susceptibility models

**DOI:** 10.18632/aging.100767

**Published:** 2015-06-24

**Authors:** Jian-Hua Mao, Antoine M. Snijders

**Affiliations:** Life Sciences Division, Lawrence Berkeley National Laboratory, Berkeley, CA, USA

While the biological effects of low-dose radiation exposure are increasingly better understood, continued research is needed to develop better individual risk-based models. Human population exposures to low doses of ionizing radiation (defined by the US Department of Energy as less than 10 rem or 0.1 Sv) are increasing due to occupational exposures of nuclear plant workers, waste managers and medical workers, and increasing use in security screening and medical diagnostic and therapeutic procedures. The annual dose to the American population has doubled over the last 20 years [1]. This is causing growing concerns in many communities since the potential adverse health effects of low-dose radiation exposure are not well understood. Currently, risk predictions are based on a one-size-fits- all linear-no-threshold model (the LNT model). This model predicts that (cancer) risk from radiation exposure is linearly related to exposed dose. While this model may be appropriate for risk predictions at high doses, the shape of the dose response curve at low doses is not well understood. We, and others, have provided evidence against the LNT risk model in the low-dose region of exposure and have shown that low-dose radiation responses appear to be strongly influenced by genetic makeup [2].

Clearly, epidemiological data are important and suggest that low dose exposures can increase cancer risk, but existing data remains controversial [3]. However, detecting genetic variants that dictate the low-dose radiation response in human populations is fraught with difficulties. The genetic heterogeneity in humans necessitates the collection of large numbers of DNA samples from patients and control populations, with no guarantee that the methods presently available would allow the detection of the most important genetic variations. Furthermore, the host environment (including other exposures, diet, lifestyle, etc) and individual health status are likely to play a determining role in the low-dose radiation response, but are difficult to control in human populations. On the other hand, parallel studies in mice offer many advantages for the study of the genetic basis of complex traits. These include: well-designed populations for a specific question, standardized husbandry, comprehensive analysis of phenotypes and the ability to manipulate the system. Furthermore, to recapitulate genetic hetero-zygosity of humans, two powerful genetically distinct mouse resources, Diversity Outbred (DO) and Collaborative Cross (CC) mice, have been generated by the mouse research community [4, 5]. DO and CC mice will enable researchers to rapidly map genetic loci at high resolution and identify individual genes involved in disease, such as cancer.

Radiation exposure can affect epithelial cells, which are at risk for developing cancer, and stromal cells (e.g. the tissue microenvironment and immune system), which not necessarily become malignant, but can influence tumor processes in epithelial cells [6], both of which contribute to radiation carcinogenesis. We recently used mouse models to study the contribution of host genetic variations that affect stromal microenvironments and systemic responses in cancer risk after exposure to low-dose ionizing radiation [7]. We transplanted unirradiated Trp53−/− mammary epithelial cells into the mammary glands of genetically diverse mice that had been irradiated three days prior to transplantation. We observed that low-dose radiation exposure reduced mammary tumor development in this genetically diverse mouse population. We found two genetic loci associated with tumor latency in un-irradiated control mice. On the other hand, we identified fifteen genetic loci that control tumor latency in low-dose irradiated mice. Since only the host was irradiated, and not the cells that eventually developed into a tumor, these genetic loci control tumor latency through the host (systemically and stromally) and provide strong evidence for the interaction between our environment and host genetics in controlling risk.

These and studies including CC and DO genetically diverse mouse resources are crucial for identifying genes that drive radiation-induced health effects and individual susceptibilities. This information together with fundamental mechanistic studies will permit more biologically realistic mathematical models to be developed to extend risk evaluation to dose levels below which direct human data are unavailable and will provide regulatory agencies, such as DOE, with the necessary data to develop accurate regulatory standards for exposure to low dose radiation.
